# Analysis of the oligomeric states of nucleophosmin using size exclusion chromatography

**DOI:** 10.1038/s41598-018-22359-w

**Published:** 2018-03-05

**Authors:** Gyosuke Sakashita, Hitoshi Kiyoi, Tomoki Naoe, Takeshi Urano

**Affiliations:** 1 Department of Biochemistry, Shimane University School of Medicine, Izumo, 693-8501 Japan; 2 Department of Hematology and Oncology, Nagoya University Graduate School of Medicine, Nagoya, 466-8550 Japan; 3 National Hospital Organization Nagoya Medical Centre, Nagoya, 460-0001 Japan

## Abstract

Nucleophosmin (NPM1) is a multifunctional phosphoprotein which plays important roles in diverse biological processes. NPM1 can form homo- or hetero-oligomers through its N-terminal region, and bind DNA and RNA through its C-terminal region. However, the monomer-oligomer distribution of NPM1, and the extent of NPM1 binding and unbinding to RNA in living cells, are not fully understood. In this work, we analysed molecular complexes of NPM1 using size exclusion chromatography. We found that a substantial fraction of NPM1 behaves as an oligomer in HeLa cells. Furthermore, we identified three distinct oligomeric states of NPM1 using molecular characterization techniques such as subcellular localization and RNA binding. Finally, we found that heterozygous expression of a leukemia-associated NPM1 mutant significantly decreases the RNA binding level. Our data demonstrate that size exclusion chromatography provides a powerful tool for analysing NPM1 oligomers.

## Introduction

Nucleophosmin (NPM1, also known as B23, NO38, or numatrin) was originally identified as a highly phosphorylated protein in the nucleolus^1^. NPM1 also localizes to the nucleoplasm, and shuttles between the nucleus and the cytoplasm^2,3^. NPM1 is involved in various biological processes such as ribosome biogenesis^4–7^, centrosome duplication^3^, genome instability^8^ and apoptosis^9,10^.

NPM1 forms homo- or hetero-oligomers through its N-terminal region^11,12^. Disruption of NPM1 oligomers by RNA aptamers results in mislocalization of NPM1 and apoptosis in cancer cells, indicating the importance of NPM1 oligomers^13^. The C-terminal region of NPM1 comprises two functional domains. One is the nucleic acid-binding domain^14^ that reportedly binds the G-quadruplex structure of rDNA^15^, the promoter region of the *superoxide dismutase 2* (*SOD2*) gene^16^, and 28S, 5.8S, and 5S rRNA in living cells^17,18^. The second C-terminal domain is a nucleolar localization signal domain^19^. These two domains are suggested to be structurally related^20^.

The overexpression of NPM1 has been observed in many types of solid tumors, including gastric^21^, prostate^22^, liver^23^ and colon^24^. Translocation in the *NPM1* gene has been reported for several hematopoietic malignancies^25,26^; for example, t(2;5)(p23;q35) in 75% of anaplastic lymphoma kinase-positive anaplastic large cell lymphoma^27^, t(5;17)(q35;q31) in less than 1% of acute promyelocytic leukemia^28^, and t(3;5)(q25;q35) in less than 1% of acute myeloid leukemia (AML)^26^. Furthermore, approximately one-third of AML patients harbor frameshift mutations in exon 12 of the *NPM1* gene^29^, resulting in the generation of a nuclear export signal in the C-terminal region of NPM1 and localization of the mutant NPM1 (NPM1c) to the cytoplasm.

Most proteins play physiological roles through temporal interactions with other molecules, or as stable molecular complexes with other proteins, possibly as homo- or hetero-oligomers. Oligomeric proteins exist in at least two different states: monomer and oligomers. A technique is therefore required for isolating monomers and oligomers in order to investigate functional differences between these states. The functions of both N-terminal and C-terminal regions of NPM1 are well-studied. In contrast, details regarding the monomer-oligomer distribution of NPM1 are not fully understood and little is known regarding the extent of NPM1 binding and unbinding to RNA in living cells.

In this study, we isolated NPM1 oligomers and monomer using size exclusion chromatography (SEC) and Western blotting. We show that a substantial fraction of NPM1 in living cells behaves as at least three distinct oligomeric states with different characteristics, such as localization and binding to RNA. Our data demonstrate that the combination of SEC and Western blotting provides a powerful tool for investigating NPM1 oligomers.

## Results

### Specificity of anti-NPM antibodies

**Figure 1:**
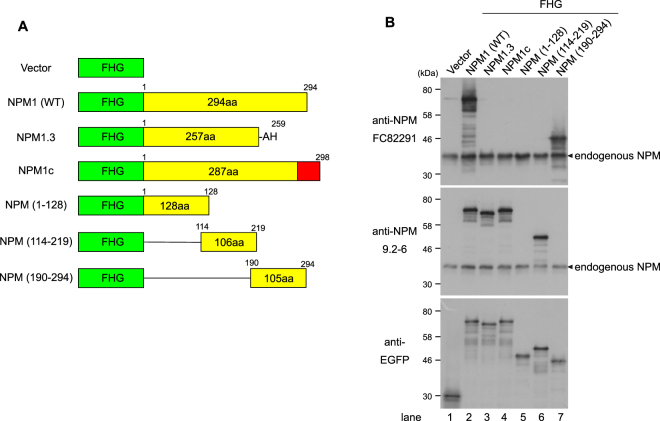
Specificity of anti-NPM antibodies. (**A**) Diagram of the FHG (FLAG-HA-EGFP)-NPM constructs. The amino acid sequences that constitute wild type NPM1 are represented by yellow. NPM1.3: a splice variant of NPM1, lacking the 37 C-terminal amino acids of NPM1, NPM1c: an AML patient-related NPM1 mutant. The 11 C-terminal amino acids which are substituted by frameshift mutation are represented by red. (**B**) 293T cells were transfected by the expression vectors described in A. After two days, the cells were lysed with SDS-sample buffer and analysed by immunoblot using commercially available anti-NPM (clone FC82291), anti-NPM which we raised previously (clone 9.2–6), or anti-EGFP.

In this study, we used two antibodies that recognize NPM1. One is a commercially available antibody against NPM, clone FC82291. According to the information provided by the supplier, the epitope of FC82291 lies within the 68 amino acids of the C-terminus of NPM1. The other antibody is the clone 9.2–6, which we raised previously^30^. To obtain more information about the epitope of NPM antibodies, we transfected an expression vector encoding NPM and its deletion mutants tagged with FHG (FLAG, HA and EGFP linked to the N-terminus) to 293T cells (Fig. 1A). As shown in Fig. 1B, the antibody FC82291 recognized NPM1 but did not recognize NPM 1.3, a splice variant of NPM1 which lacks the 37 C-terminal amino acids of NPM1, nor did it recognize NPM1c, an AML patient-related NPM1 mutant in which the 7 C-terminal amino acids of NPM1 are substituted to 11 unrelated amino acids by a frameshift mutation (Fig. 1B, lanes 2–4). Therefore, we used the commercially available antibody FC82291 as an NPM1-specific antibody. On the other hand, the antibody clone 9.2–6 recognized NPM (114–219), a central region of NPM1, as well as full length NPM1, NPM1.3 and NPM1c (Fig. 1B).

### Separation of NPM1 by size exclusion chromatography (SEC)

**Figure 2:**
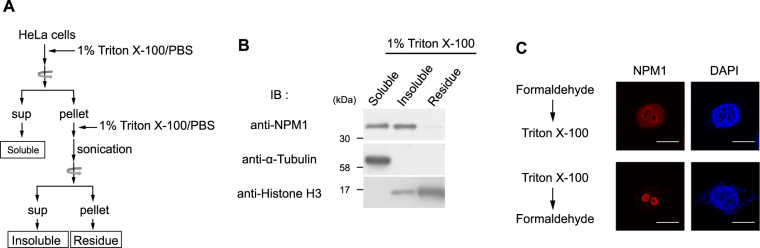
Distribution of NPM1 by simple cell fractionation. (**A**) Summary of the process of cell fractionation. (**B**) Distribution of NPM1 in the sample prepared in A. (**C**) Immunofluorescence of endogenous NPM1. (*Upper panel*) HeLa cells were fixed with formaldehyde, then permeabilized using Triton X-100. (*Lower panel*) HeLa cells were permeabilized using Triton X-100, then fixed with formaldehyde. Scale bar, 10 μm.

**Figure 3:**
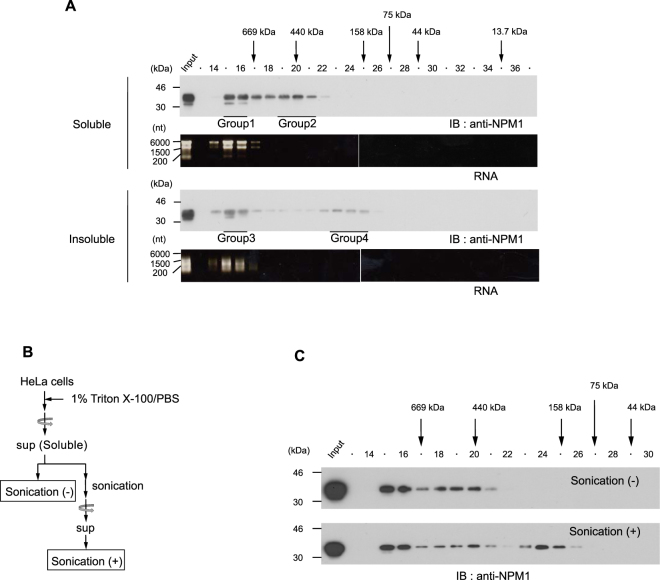
Elution profiles of endogenous NPM1 in HeLa cells. (**A**) (*Upper panel*) Elution profiles of Triton X-100 soluble NPM1 and RNA. (*Lower panel*) Elution profiles of Triton X-100 insoluble NPM1 and RNA. Endogenous NPM1 was detected by immunoblot. Protein mass standards are indicated above the *panel*: thyroglobulin (669 kDa), ferritin (440 kDa), aldolase (158 kDa), conalbumin (75 kDa), ovalbumin (44 kDa), and Ribonuclease A (13.7 kDa). RNA was detected by ethidium bromide staining. The four NPM1 groups (Group 1 to Group 4) identified by SEC are underlined. (**B**) Schematic representation of sample preparation in C. (**C**) Elution profile of sonication-treated Triton X-100 soluble NPM1 prepared in B.

We first performed simple lysis and fractionation of HeLa cells using Triton X-100 (Fig. 2A). Approximately half of the total NPM1 protein was solubilized using these conditions and half remained insolubilized. This ‘insoluble NPM1’ was retained in the cell pellet following the first centrifugation and was subsequently solubilized by sonication (Fig. 2B). As shown in Fig. 2C, NPM1 localizes at both the nucleoplasm and the nucleolus, as determined by immunostaining involving permeabilization with Triton X-100 followed by cell fixation with formaldehyde (Fig. 2C, *upper panel*). Conversely, when cells were permeabilized using Triton X-100 prior to fixation to remove the soluble NPM1, no nucleoplasmic NPM1 was detected while nucleolar NPM1 remained (Fig. 2C, *lower panel*). These results suggest that biochemically fractionated ‘soluble NPM1’ and ‘insoluble NPM1’ mainly represent nucleoplasmic NPM1 and nucleolar NPM1, respectively. Therefore, we analysed soluble NPM1 and insoluble NPM1 by SEC independently. The elution profile of soluble NPM1 from asynchronous HeLa cells showed two peaks, one in fractions 15 to 16 (Group 1), and one in fractions 19 to 21 (Group 2), suggesting that there are at least two populations of nucleoplasmic NPM1 complexes (Fig. 3A). The elution profile of insoluble NPM1 was different from that of soluble NPM1. Whereas the peak in fractions 15 to 16 (Group 3) is also detected in the profile of soluble NPM1, the peak in fractions 23 to 25 (Group 4) was only observed upon fractionation of insoluble NPM1. We found that most of the RNA, which probably largely comprises rRNA, was detected as a single peak in fractions 15 to 16 of both soluble and insoluble cell lysates following SEC. The observed peaks in both the soluble and insoluble fractions coincided with the higher molecular weight peak obtained from the NPM1 fractions (Group 1 and Group 3). RNA molecules in the insoluble fractions were fragmented to some extent, probably due to sonication. Therefore, we suspected that Group 4 NPM1 was a result of sonication. To test this possibility, soluble cell lysates typically prepared without sonication in this study were used to examine the effects of sonication on the elution profiles obtained by SEC (Fig. 3B). When the soluble cell lysates were subjected to SEC without sonication, no peak was observed in fractions 23 to 25 (Fig. 3C, *upper panel*), whereas sonication of the soluble cell lysates resulted in a peak in fractions 23 to 25, as observed for insoluble NPM1. Therefore, insoluble Group 4 NPM1 detected in fractions 23 to 25 may be an artifact resulting from sonication. These data suggest that nucleolar NPM1 originally provides a single peak in fractions 14 to 16 upon SEC, although NPM1 may not be fully intact due to sonication.

### Association of NPM1 with RNA in high molecular weight fractions

**Figure 4:**
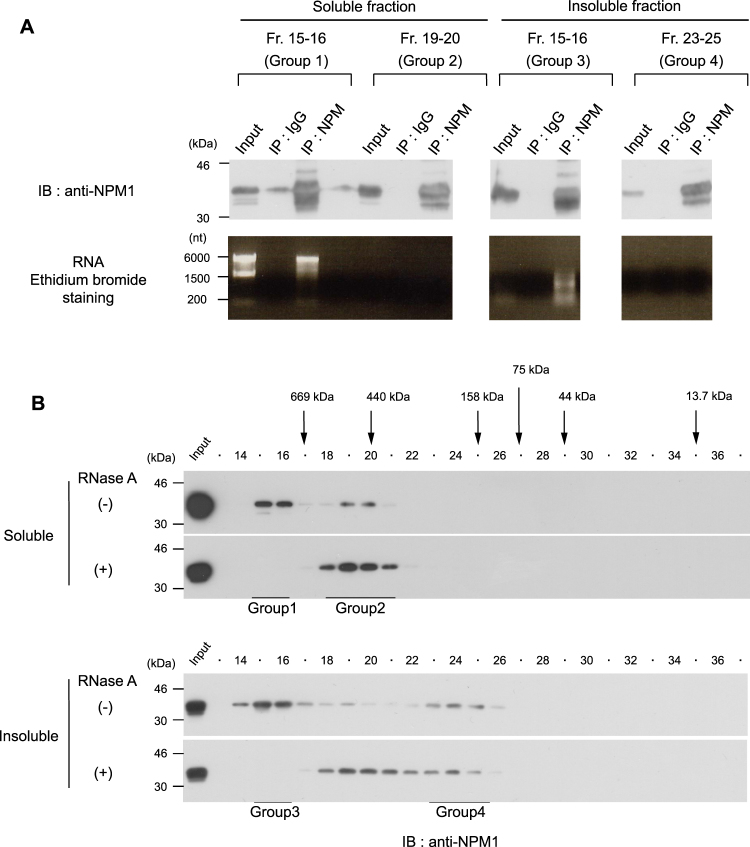
Association of NPM1 with RNA in the high molecular weight fractions. (**A**) Triton X-100 soluble and insoluble cell lysates were subjected to SEC. The indicated fractions were pooled, then immunoprecipitated with anti-NPM. NPM1 and RNA were detected by immunoblot and ethidium bromide staining, respectively. (**B**) HeLa cell lysates prepared as in Fig. 2A were treated with or without RNase A, then subjected to SEC. Fractions containing the four NPM1 groups identified in Fig. 3A are underlined.

Since NPM1 is an rRNA binding protein, we determined which group of NPM1 associates with RNA. We first used the commercially available monoclonal antibody FC82291. We could not detect the association of NPM1 with RNA using this NPM1 specific antibody (Supplementary Figure 1). The epitope of the antibody is located at the C-terminal end of NPM1, overlapping with the RNA-binding domain of NPM1. These results suggest that the antibody inhibits NPM1 binding to RNA in a competitive manner. Therefore, to investigate the association between NPM1 and RNA, we used the anti-NPM monoclonal antibody 9–2.6, which recognizes the central region of NPM1^30^. As expected, RNA coprecipitated with the Group 1 and Group 3 fractions of NPM1 (Fig. 4A). Next, we investigated the extent to which NPM1 associates with RNA in fractions 15 and 16. To this end, the soluble and the insoluble cell lysates were treated with RNase A prior to analysis by SEC. As shown in Fig. 4B, after RNase A treatment, Group 1 NPM1 (fractions 15 and 16) was completely shifted to fractions corresponding to Group 2 NPM1 (fractions 19 and 20). Similarly, Group 3 NPM1 (fractions 14 to 16) also completely shifted to fractions 19 and 20. These results suggest that almost all of Group 1 and Group 3 of NPM1 associate with RNA in either a direct or indirect manner, and that the majority of cellular NPM1 elutes in fractions 18 to 20 when RNA is removed from the NPM1 complex.

### A substantial fraction of NPM1 is oligomeric in cells

**Figure 5:**
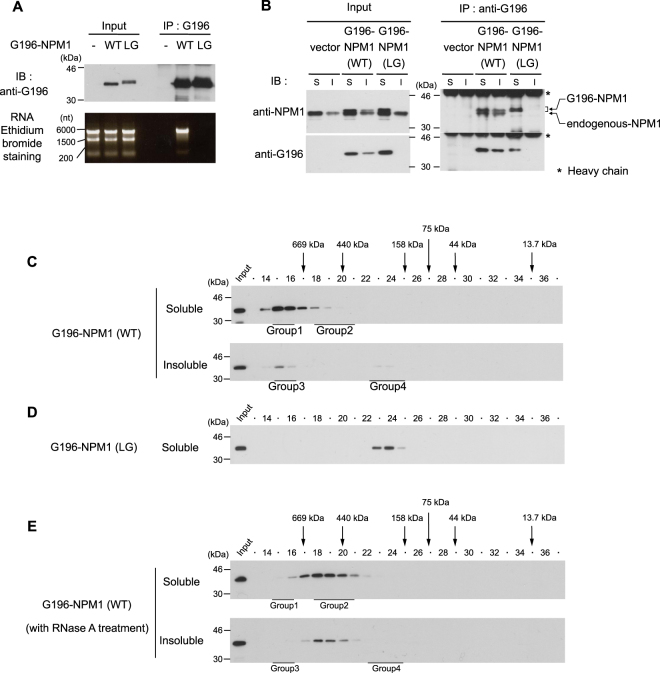
Elution profiles of NPM1 monomer and oligomers expressed in cells. 293T cells were transfected with expression vector for G196-NPM1 (WT) or G196-NPM1 (LG). (**A**) The Triton X-100 soluble lysates were used for immunoprecipitation with anti-G196 antibody. G196-NPM1 and extracted RNA were detected by immunoblot and ethidium bromide staining, respectively. (**B**) Interaction of G196-NPM (WT) or G196-NPM1 (LG) with endogenous NPM1. The Triton X-100 soluble (S) and insoluble (I) cell lysates were treated with RNase A, then immunoprecipitated with anti-G196 antibody. (**C**) Elution profiles of G196-NPM1 (WT) in the Triton X-100 soluble and insoluble cell lysates. Fractions containing the four NPM1 groups identified in Fig. 3A are underlined. (**D**) Elution profile of G196-NPM1 (LG) in the Triton X-100-soluble cell lysates. (**E**) Elution profiles of G196-NPM1 (WT) in the RNase A-treated Triton X-100 soluble and insoluble cell lysates. Fractions containing the four NPM1 groups identified in Fig. 3A were underlined.

A previous study demonstrated that the bacterially expressed N-terminal region of NPM forms pentamers^12^, and the ability of NPM1 to form oligomers has been estimated through the interaction between exogenously expressed NPM1 and endogenous NPM1^31^. To gain insight into the oligomeric status of cellular NPM1, we introduced cDNAs for wild type NPM1 and LG mutant (L102A and G105A) NPM1 which does not form oligomers^31^ into 293T cells. Both cDNAs were fused to the G196 tag sequence encoding five amino acids^32^. When cells were lysed with Triton X-100-containing buffer, RNA was coprecipitated with G196-NPM1 (WT), but not with G196-NPM1 (LG) (Fig. 5A). In order to detect NPM1-NPM1 interaction independent of RNA, we next performed immunoprecipitation using RNase A-treated cell lysates. Whereas G196-NPM1 (WT) was coprecipitated with endogenous NPM1 in both the soluble and the insoluble cell lysates, G196-NPM1 (LG) was not coprecipitated with endogenous NPM1 (Fig. 5B), confirming the results of the previous report^31^. These results suggested that at least a part of the G196-NPM1 (WT) population exists as oligomers and that G196-NPM1 (LG) exclusively exists as monomers in cells.

**Figure 6:**
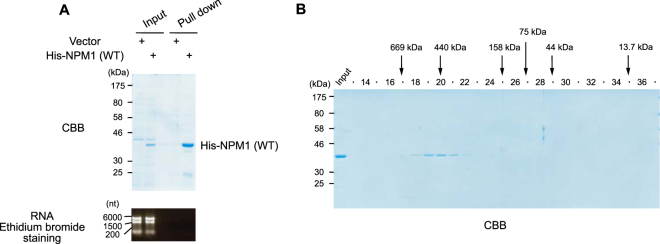
Purified recombinant NPM1 shows a similar elution profile to that of RNase A-treated cellular NPM1. (**A)** Bacterially expressed His-TEV-NPM1 was purified as described in experimental procedures. Purified proteins showed high purity and did not contain any RNA. (**B)** Elution profile of purified His-TEV-NPM1 proteins.

**Figure 7:**
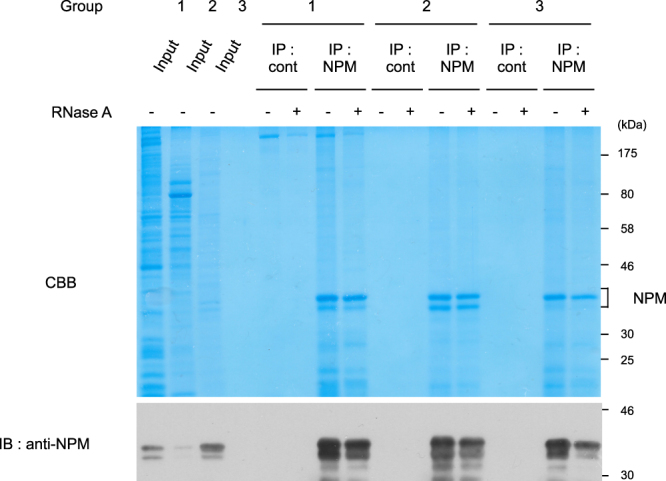
The main component of cellular NPM1 complex is NPM1 and its variant. Both the Triton X-100 soluble and insoluble lysates from HeLa cells were subjected to SEC. Fractions 15 and 16 from the soluble sample (for Group 1), fractions 19 to 21 from the soluble sample (for Group 2), and fractions 15 and 16 from the insoluble sample (for Group 3) were separately pooled. After treatment with or without RNase A, each pooled sample was immunoprecipitated with anti-NPM antibody. Proteins were detected by CBB staining (*Upper panel*) and immunoblot using anti-NPM antibody (*Lower panel*).

Next, we performed SEC by using cell lysates expressing G196-NPM1 (WT) or G196-NPM1 (LG) without RNase A treatment. G196-NPM1 (WT) was eluted in fractions corresponding to Group 1 and Group 3, suggesting that the majority of G196-NPM1 (WT) in both the soluble and the insoluble cell lysates associates with RNA, making it difficult to distinguish oligomers from monomers (Fig. 5C). On the other hand, monomeric G196-NPM1 (LG), which was not coprecipitated with RNA, showed a single peak in fractions 23 and 24 on SEC (Fig. 5D). To exclude the influence of RNA on the elution profile of G196-NPM1 (WT), the cell lysates were treated with RNase A prior to SEC. In the absence of RNA, G196-NPM1 (WT) was eluted in fractions 18 to 20 in both the soluble and the insoluble samples (Fig. 5E), consistent with the case observed in HeLa cells (Fig. 4B). Since G196-NPM1 (WT) forms oligomers, this result raises the possibility that oligomeric NPM1 is eluted in fractions 18 to 20 on SEC. To investigate the elution profile of purified NPM1 oligomers, we prepared bacterially expressed His-tagged NPM1 (WT) which is known to form oligomers. His-tagged NPM1 (WT) also possesses the ability to bind bacterial RNA, and thus the cell lysates were treated with RNase A. Purified His-tagged NPM1 (WT) protein showed a purity of more than 90% as determined by Coomassie Brilliant Blue (CBB) staining of the gel, and did not contain RNA (Fig. 6A). The elution profile of purified His-tagged NPM1 (WT) showed a single peak in fractions 18 to 20 (Fig. 6B), consistent with that of RNase A-treated G196-NPM1 (WT) expressed in 293T cells. To examine whether cellular NPM1 bind to other proteins with similar stoichiometry to NPM1, each group of NPM1 which was separated by SEC was immunoprecipitated either with or without RNase A treatment. We found one or two bands, which can be detected by CBB staining between 30 kDa and 46 kDa, as the major component(s) in the immunoprecipitates of all groups (Fig. 7, *upper panel*). Western blot analysis using anti-NPM antibody strongly suggested that these bands are NPM1 and its variants (Fig. 7, *lower panel*). Taken together, these results suggest that a substantial fraction of NPM1 is oligomeric in cells.

### Association of NPM1 with RNA is attenuated in a NPM1c-positive cell line

**Figure 8:**
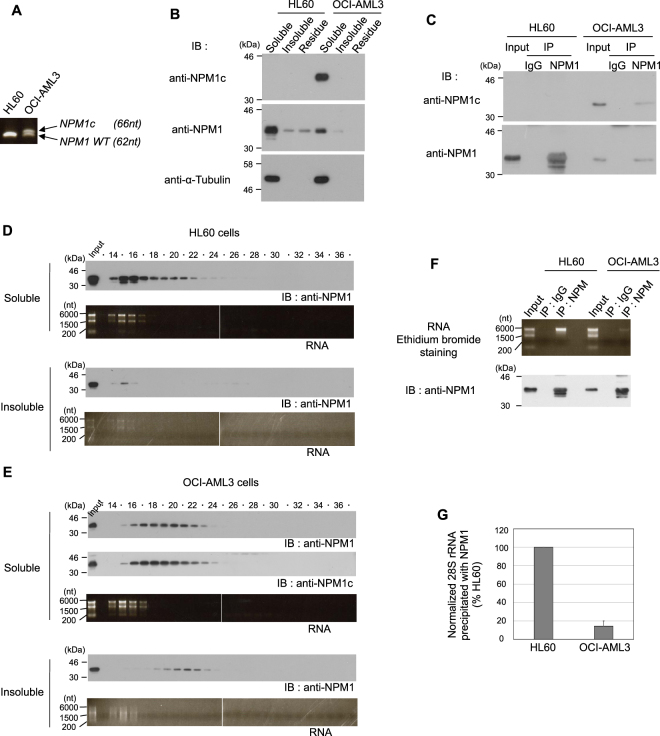
The association of NPM1 with RNA is attenuated in a NPM1c-positive cell line. (**A**) RNA was extracted from HL60 (expressing *NPM1 (WT)*) and OCI-AML3 (expressing *NPM1 (WT)* and *NPM1c*) cells. The expression of *NPM1 (WT)* and *NPM1c* was detected by RT-PCR. (**B**) Distribution of NPM1 in HL60 and OCI-AML3 cells prepared as in Fig. 2A. Then, NPM1 (WT) and NPM1c were detected by Western blotting. (**C**) HL60 and OCI-AML3 cells were lysed with buffer containing Triton X-100, SDS, and DOC. The lysate was immunoprecipitated with antibody against NPM1. NPM1 (WT) and NPM1c were detected by Western blotting with the indicated antibodies. (**D,E**) Triton X-100 soluble and insoluble lysates prepared from HL60 (**D**) and OCI-AML3 (**E**) cells were subjected to SEC. NPM1 (WT) and NPM1c were detected by Western blotting. RNA was extracted from each fraction and detected by ethidium bromide staining. (**F)** Interaction of NPM1 complex with RNA. The Triton X-100 soluble lysates from HL60 and OCI-AML cells were immunoprecipitated with anti-NPM antibody. NPM1 and extracted RNA were detected by immunoblot and ethidium bromide staining, respectively. (**G**) Quantification of the data from panel F. The normalized RNA levels coprecipitated with NPM1 are shown. The data represent the average of three independent experiments ± SEM for each condition.

Approximately one third of AML patients carry frameshift mutations in exon 12 of the *NPM1* gene in one allele. NPM1c, the protein product from the mutant allele, has several amino acid substitutions near the C-terminal end of NPM1, and this area constitutes part of the RNA-binding domain. NPM1c has biological characteristics distinct from wild type NPM1, such as a defective nucleolar localization signal and the appearance of a nuclear export signal^19,33^. Therefore, we investigated whether the expression of NPM1c has any effect on the SEC elution profile of wild type NPM1 expressed from the normal allele. To this end, we used two AML cell lines: HL60 cells, which express wild type NPM1, and OCI-AML3 cells, a known NPM1c-positive cell line. Since HL-60 cells express only wild type *NPM1*, a single band of 62 nt was observed by RT-PCR (Fig. 8A, left lane). On the other hand, OCI-AML3 cells express both wild type *NPM1* and *NPM1c*. Therefore, two bands, one of 62 nt (wild type *NPM1*) and one of 66 nt (*NPM1c*, which contains four nucleotide insertions), were detected by RT-PCR (Fig. 8A, right lane). The RNA level of *NPM1c* and wild type *NPM1* in OCI-AML3 cells was shown to be almost equal. Western blot analysis revealed that wild type NPM1 mainly shows Triton X-100 soluble in both cell lines (Fig. 8B). On the other hand, NPM1c was detected only in the soluble fraction in OCI-AML3 cells. The expression of wild type NPM1 in OCI-AML3 cells was lower than that of HL60 cells (Fig. 8B, *middle panel*). Given that there is no mutation in the amino acid sequence of the N-terminal oligomerization domain in NPM1c, NPM1c is expected to form an oligomer with NPM1 (WT) in OCI-AML3 cells. Therefore, we performed an immunoprecipitation assay using the antibody FC82291 against NPM1. This antibody did not recognize NPM1c because its epitope is the C-terminus of NPM1 (WT) (Fig. 1B, *upper panel*, lane 4). As expected, NPM1c coprecipitated with NPM1 (WT), demonstrating that NPM1c forms an oligomer with NPM1 (WT) in OCI-AML3 cells (Fig. 8C). The elution profiles of Triton X-100 soluble and insoluble NPM1 from HL60 cells are similar to the results obtained using HeLa cells (Fig. 8D). In contrast, the elution profiles of wild type NPM1 from OCI-AML3 cells was quite different from that of HL60 cells. Triton X-100 soluble NPM1 (WT) was detected as a relatively broad peak in fractions 16 to 23 (Fig. 8E). NPM1c also eluted as a broad peak, but the peak fractions were slightly different from those of NPM1 (WT). Triton X-100 insoluble NPM1 (WT) was detected in fractions 20–22. Notably, neither peak coincided with the peak due to RNA, which mainly eluted in fractions 15 and 16. To investigate whether wild type NPM1 associates with rRNA in OCI-AML3 cells, we performed an immunoprecipitation assay using Triton X-100 soluble cell lysates. As shown in Fig. 8F, the level of RNA which coprecipitated with NPM1 in OCI-AML3 cell lysate was significantly lower compared with that of HL60 lysate (Fig. 8G). Taken together, these results indicate that most wild type NPM1 does not bind with rRNA in OCI-AML3 cells.

## Discussion

In this study, we used SEC to analyse the molecular complexes formed by NPM1. SEC can be performed under a wide range of experimental conditions such as pH, salt concentration, and detergents, depending on the purpose of the experiment. In addition, SEC also permits the use of subsequent processes, such as enzymatic reaction and immunoprecipitation. This flexibility of the SEC technique prompted us to investigate two important states of NPM1: the NPM1 oligomers, and the NPM1/rRNA complex.

We showed that a substantial fraction of NPM1 is oligomeric in HeLa cells upon SEC, indicating that the major function of NPM1 is exhibited as NPM1 oligomers. Therefore, it may be necessary to take other components of NPM1 oligomers into account in order to understand the role of NPM1 itself. Two splice variants, named NPM1.3 (UniProt P06748–3) and NPM1.2 (UniProt P06748–2), have been reported to date^34^. NPM1.3 lacks the 37 C-terminal amino acids of NPM1, resulting in complete loss of nucleic acid binding ability^14^, and NPM1.2 lacks 29 amino acids (residues 195–223) of NPM1. Although NPM1.2 retains RNA binding ability, its affinity has been reported to be lower than that of NPM1^18^. Given that both isoforms can form oligomers with NPM1 through the conserved N-terminal oligomerization domain, the function of NPM1 oligomers may be dependent on the composition of the NPM1 isoforms.

Approximately equal levels of NPM1 monomer and oligomer in cultured cell lines were detected in a previous study^35^. In this study, NPM1 monomer and oligomer were isolated by ether native PAGE, SDS-PAGE without heat treatment of the protein samples, or SDS-PAGE using crosslinked protein samples. However, it is difficult to compare the levels of proteins differing significantly in molecular weight. As discussed below, we found that approximately half of the NPM1 molecules form a complex with 28S rRNA and the molecular weight of the NPM1/28S rRNA complex might be too high to migrate in a polyacrylamide gel. Furthermore, large differences in molecular weight usually lead to different transfer efficiencies of proteins from gel to membrane upon blotting; consequently, the amount of NPM1 oligomer might be underestimated compared with NPM1 monomer following native PAGE or SDS-PAGE using crosslinked protein samples. The method which combined SEC and Western blotting separated NPM1 monomer and oligomer into different fractions using non-denaturing conditions by SEC, followed by detection as monomer by Western blotting under denaturing conditions. Therefore, the result obtained using this method is likely more accurate.

We found that there are at least three groups of NPM1 oligomers (Group 1–3). Group 1 NPM1 localizes in the nucleoplasm, and forms a complex with rRNA. Group 2 NPM1 also localizes in the nucleoplasm but does not bind with rRNA. Group 3 NPM1 localizes in the nucleolus and is associated with rRNA. Given that NPM1 is a non-ribosomal protein involved in ribosome biogenesis, these three groups of NPM1 might be involved in different stages of ribosome biogenesis. On the other hand, many proteins not involved in ribosome biogenesis have been reported to bind with NPM1^36^. NPM1 complex which contains these binding proteins may also be included in Group 2 at a lower level, since the major component of NPM complex is NPM1 and its variants. We also found that nucleolar-localized Group 4 NPM1, which was solubilized by sonication of the cell lysate, showed similar peak fractions to that of monomeric NPM1 (LG). However, we cannot exclude the possibility that Group 4 NPM1 is due to sonication of the sample, since monomer-like NPM1 was observed when nucleoplasmic NPM1 oligomer was subjected to additional sonication. Further studies are thus required.

The elution peaks of G196-NPM1 monomer and oligomer isolated by SEC from cells corresponded to masses of approximately 158 kDa (Fig. 5D) and 500 kDa (Fig. 5E), respectively, and deviate from the calculated molecular weight of NPM1. Similar conflicting results were obtained using bacterially expressed NPM1 (Fig. 6B). These discrepancies between observed and predicted molecular weight might result from the structure of NPM1. The N-terminal domain of NPM1 is in a highly ordered conformation to form oligomers^12,37^, the central region is suggested to be disordered^18^, and the C-terminal region of NPM1 consists of three-helix bundles^20^. Therefore, the structures of NPM1 monomer and oligomers might result in an elution profile different from that of a typical globular protein with a molecular weight similar to NPM1.

The AML-associated mutation of NPM1 occurs in a heterogeneous manner and therefore both NPM1 (WT) and NPM1c express in OCI-AML3 cells. Recent studies have demonstrated that NPM1c loses its binding ability to the G-quadruplex structure of DNA *in vitro*^15^ and gains the nuclear export signal (NES), which is a well-known difference between NPM1 (WT) and NPM1c. These results suggest that NPM1c also fails to bind RNA. In support of this idea, we found that the elution profile of NPM1c does not coincide with that of RNA. Surprisingly, the elution profile of NPM1 (WT) also did not coincide with that of RNA. Indeed, the amount of rRNA coprecipitated with NPM1 was greatly attenuated in OCI-AML3 cells. These results indicate that ribosomes expressed in OCI-AML3 cells do not require interaction between NPM1 and rRNA during maturation. These abnormalities might be related to the pathology of NPM1c-positive AML. The reason why even NPM1 (WT) failed to bind rRNA in OCI-AML3 cells could be due to two factors related to NPM1 oligomers. The first factor is an NES signal in NPM1c. NPM1c has been reported to mainly localize to the cytosol. When NPM1 (WT) forms an oligomer with NPM1c, the oligomer might localize to the cytosol, losing the opportunity to bind rRNA in the nucleus where ribosome biogenesis takes place. The second factor is the ratio of NPM1c to NPM1 (WT) in the NPM1 oligomer. A previous study reported that NPM1 oligomer exhibited decreased binding ability to rRNA when the ratio of NPM1.3 to NPM1 (WT) was increased in an *in vitro* reconstitution assay^38^. Based on this observation, increasing the ratio of NPM1c in NPM1 oligomers might result in decreased binding ability of NPM1 oligomers to rRNA.

Our study indicates that it is important to reveal the functional role of NPM1 oligomers, and that SEC provides a powerful tool for analysing NPM1 oligomeric states.

## Methods

### Antibodies

Mouse monoclonal antibodies against NPM1 (clone FC82291, B0556, Sigma-Aldrich, St. Louis, MO, USA), α-tubulin (T6199, Sigma-Aldrich), β-actin (A5441, Sigma-Aldrich) and rabbit polyclonal antibodies against GFP (60–011, BioAcademia, Osaka, Japan) and histone H3 (#9715, Cell Signaling, Danvers, MA, USA), were purchased commercially. Mouse monoclonal antibodies against NPM (clone 9–2.6)^30^ and G196^32^ were described previously. Rabbit polyclonal antibody against NPM1c was raised using a synthetic peptide corresponding to the C-terminal sequence of NPM1c type A (DLCLAVEEVSLRK). Horseradish peroxidase-conjugated goat F(ab’)_2_ anti-mouse IgG (H+L) (#710–133, Rockland Immunochemicals, Limerick, ME, USA), goat anti-rabbit IgG (111–035–003, Jackson ImmunoResearch Laboratories, West Grove, PA, USA) and Alexa 594-conjugated goat anti-mouse IgG (H+L) (A-11032, ThermoFisher Scientific, Waltham, MA, USA) were purchased commercially.

### Plasmid

All primers used in this study are listed in Table S1. The cDNA for FLAG-HA-EGFP (FHG) was generated using pEGFP-C1 vector (Takara Bio, Shiga, Japan) as the template. The PCR product was digested with *Bgl*II and *Eco*RI and subcloned into a pBSK-*Bgl*II vector, which is a modified version of pBluescript II SK (+) vector (Agilent Technologies, Santa Clara, CA, USA) and harbors the *Bgl*II recognition sequence in its multiple cloning site. To construct the FHG expressing vector, the *Bgl*II-*Eco*RI fragment of FHG cDNA digested from FHG/pBSK-*Bgl*II vector was inserted into the *Bam*HI-*Eco*RI sites of pcDNA3.1 (+) vector (ThermoFisher Scientific). To construct G196/pcDNA3 vector, an annealed fragment of the G196 oligonucleotides was inserted into pcDNA3.1 (+) vector which was digested with *Hind*III and *Xho*I.

The cDNA sequences of NPM1 (WT), NPM1.3 and NPM1c were PCR amplified using oligo(dT)-primed first-strand cDNAs from the HeLa and OCI-AML3 cell lines. The cDNA for NPM deletion mutants was amplified by PCR using the cDNA for NPM1 (WT) as template. The cDNA for NPM1 (LG) mutant was generated by overlap PCR using the internal primers in addition to NPM1 sense primer and NPM1 antisense primer.

The *Bam*HI-*Eco*RI fragment of cDNA for NPM was inserted into FHG/pcDNA3.1 or G196/pcDNA3.1 and into pET28-TEV, a modified pET-28a (Novagen, Madison, WI, USA) with a hexa-histidine-tag and TEV cleavage site at the amino-terminus. The sequences of all the PCR products were confirmed.

### Cell culture, transfection

OCI-AML3 cells were obtained from DSMZ (ACC 582, Braunschweig, Germany). An ecotropic HeLa strain which expresses mCAT (a receptor for murine retrovirus), and HEK293T cells, were maintained in Dulbecco’s modified Eagle medium (Nissui, Tokyo, Japan) supplemented with 10% fetal bovine serum (Sigma-Aldrich), penicillin and streptomycin (Nacalai Tesque, Kyoto, Japan) in a 37 °C incubator with a humidified atmosphere of 5% CO_2_. HL60 and OCI-AML3 were grown in RPMI1640 medium supplemented with 10% fetal bovine serum, penicillin and streptomycin in a 37 °C incubator with a humidified atmosphere of 5% CO_2_. Transfection was performed using Lipofectamine 2000 (ThermoFisher Scientific).

### Preparation of cell lysat

To investigate the epitopes of NPM antibody, 293T cells exogenously expressing NPM were lysed with SDS-sample buffer by heating 100 °C for 3 min. For fractionation of the HeLa and 293T cells, cells were lysed with PBS containing 1% Triton X-100. For fractionation of HL60 and OCI-AML3 cells, cells were lysed with PBS containing 1% Triton X-100, 15 mM imidazole, 1 mM DTT and 40 U/ml RNase inhibitor (Takara Bio). After incubation on ice for 10 min, samples were centrifuged at 2,100 × g for 5 min at 4 °C, then the supernatant was used as the ‘Triton X-100 soluble fraction’. The resulting pellet was rinsed with lysis buffer and resuspended in new lysis buffer. The suspension was sonicated using a Handy Sonic (TOMY SEICO, Tokyo, Japan) at a power control setting of 5 for 20 sec. After centrifugation at 20,000 × g for 5 min at 4 °C, the supernatant was used as the ‘Triton X-100 insoluble fraction’. For RNase A treatment, cell lysates were treated with 40 µg/ml RNase A (Sigma-Aldrich) for 15 min at 37 °C. After centrifugation at 20,000 × g for 10 min, the supernatant was used for SEC. For interaction of NPM1 with NPM1c in OCI-AML3 cells, cells were lysed with PBS containing 1% Triton X-100, 0.1% SDS, 0.5% DOC and 15 mM imidazole.

### Size exclusion chromatography

SEC was performed using an AKTA FPLC system (GE Healthcare, Buckinghamshire, England). A Superdex 200 Increase 10/300 GL column (GE Healthcare) was equilibrated with cell lysis buffer. The column was calibrated using a gel filtration calibration kit (GE Healthcare). Each standard protein was dissolved in PBS containing 1% Triton X-100 and chromatographed on the column separately. The filtered protein samples were then fractionated on the column (0.75 ml/min; 0.5 ml/fraction).

### Immunoprecipitation and immunoblotting

Immunoprecipitation was performed using approximately 2 µg of anti-NPM, anti-G196 and anti-NPM1 for 1~3 hours. The antibodies used for immunoprecipitation were pre-conjugated with Protein G Sepharose 4 Fast Flow (GE Healthcare). After SDS-PAGE on a 12% acrylamide gel, the proteins were transferred onto an Immobilon-P membrane (Sigma-Aldrich). The membrane was blocked with 5% skim milk, then incubated with the appropriate antibodies. Blots were washed with PBS-T, and developed using an ECL-Plus system (PerkinElmer, Waltham, MA, USA). For quantitative analysis, images were acquired using ImageQuant LAS 4000 (GE Healthcare) and quantified using Multi Gauge software (Fujifilm, Tokyo, Japan).

### CBB staining of immunoprecipitated NPM1 complex

Approximately 1.4 × 10^8^ HeLa cells were fractionated as described above, and 8 ml of soluble cell lysate and 8 ml of insoluble cell lysate were prepared. Each cell lysate was separated by SEC 4 times. After SEC, fractions 15 and 16 from the soluble samples (for Group 1), fractions 19 to 21 from the soluble samples (for Group 2), and fractions 15 and 16 from the insoluble samples (for Group 3) were separately combined to provide 3 pools. Each pooled fraction was divided into four tubes for treatment with or without 40 µg/ml RNase A for 15 min at 37 °C. After centrifugation at 20,000 × g for 10 min, the supernatant was used for immunoprecipitation by anti-G196 (for negative control) or anti-NPM 9.2 antibody cross-linked to Protein G Sepharose 4 Fast Flow. Immunoprecipitates were eluted with PBS containing 1% Triton X-100 and 2 M guanidine-HCl (Nacalai Tesque). Samples were then precipitated using PAGE Clean Up Kit (Nacalai Tesque). After drying, 25 μl of SDS sample buffer was added to each sample, then heated at 100 °C for 3 min. NPM1 complex was detected in 15 μl samples by CBB staining, and 3 μl samples were used to detect NPM1 complex by Western blotting.

### Immunofluorescence

HeLa cells were grown on a glass cover coated with Type I-C Cellmatrix (Nitta Gelatin, Osaka, Japan). For the modified standard protocol, the cells were washed with PBS and fixed with PBS containing 3.7% formaldehyde for 20 min. The cells were then washed with PBS and permeabilized with PBS containing 1% Triton-X100 for 2 min. After washing with PBS, the cells were incubated with PBS containing 5% skim milk for 20 min. To remove Triton X-100 soluble proteins prior to fixation, the cells were incubated in PBS containing 1% Triton X-100 for 2 min on ice, washed twice with PBS, and then fixed with 3.7% formaldehyde for 20 min. After washing with PBS, the cells were blocked with PBS containing 5% skim milk for 20 min. Next, the cells were incubated with blocking buffer containing anti-NPM1 antibody at room temperature for 1 h. The cells were then washed with PBS and incubated with Alexa Fluor 594 goat anti-mouse IgG (1:1000) at room temperature for 20 min. After washing with PBS, the cells were mounted on glass slides using vectashield mounting medium with DAPI (Vector Laboratories, Burlingame, CA, USA). Images were acquired using confocal microscope (FV1000, Olympus, Tokyo, Japan).

### RNA extraction

For RNA extraction, 500 µl of Sepasol-RNA I Super G (Nacalai Tesque) was added to 50 µl of cell lysate, fractionated sample, or immunoprecipitated sample. For RT-PCR, 1000 µl of Sepasol-RNA I Super G was added to 2 × 10^6^ HL60 or OCI-AML3 cells. After 5 min, 100~200 µl of chloroform was added, and the mixture was centrifuged at 20,000 × g for 5 min at 4 °C. RNA extracted into the aqueous layer was precipitated by adding an equal volume of isopropanol. After rinsing with 70% ethanol, the RNA was dried and dissolved in loading buffer (43% formamide, 0.013% SDS, 0.013% bromophenol blue, 0.013% xylene cyanol, 0.013% ethidium bromide and 0.25 mM EDTA) and then treated at 70 °C for 10 min.

### Recombinant protein

B834 (DE3) *Escherichia coli* transformed with NPM1/pET28-TEV vector was grown in LB medium supplemented with kanamycin (Nacalai Tesque) at 37 °C for overnight. *E. coli* suspension (500 µl) was added to 4.5 ml of fresh LB medium and cultured at 37 °C for 1 hour. Protein expression was induced with 1mM isopropyl-β-D-1-thiogalactopyranoside (IPTG). After 2 hours’ culture, the *E. coli cells* were centrifuged at 20,000 × g for 1 min at 4 °C, then suspended in lysis buffer (PBS, 1% Triton X-100 and 15 mM imidazole). The suspension was sonicated at a power control setting of 10 for 20 sec. After centrifugation at 20,000 × g for 10 min at 4 °C, the supernatant was used for affinity purification using Ni-NTA agarose (QIAGEN, Valencia, CA, USA). After incubation at 4 °C for 1 hour, the beads were washed with lysis buffer, then treated with 40 µg/ml RNase A in lysis buffer for 15 min at 37 °C. After washing with lysis buffer, proteins were eluted using elution buffer (PBS, 1% Triton X-100 and 250 mM imidazole).

### RT-PCR analysis

Five µg of total RNA was reverse-transcribed using oligo (dT) primer and a ReverTra Ace system (TOYOBO, Osaka, Japan). The cDNAs for NPM1 (WT) and NPM1c were amplified with 25 cycles of PCR. Gel electrophoresis was performed with a 10% urea-PAGE gel using the TBE buffer system.

## Electronic supplementary material

Supplementary information
