# Supplementary material for: Analysis of the oligomeric states of nucleophosmin using size exclusion chromatography
Source: Sci Rep. 2018 Mar 5;8:4008. doi: 10.1038/s41598-018-22359-w (PMC5838202; doi:10.1038/s41598-018-22359-w)
Supplement: Supplementary file 1 — Supplementary information [file 41598_2018_22359_MOESM1_ESM.pdf]

Supplementary Material – "Analysis of the oligomeric states of nucleophosmin using size exclusion chromatography"

Gyosuke Sakashita[1], Hitoshi Kiyoi[2], Tomoki Naoe[3], and Takeshi Urano[1]

[1]Department of Biochemistry, Shimane University School of Medicine, Izumo 693-8501, Japan

[2]Department of Hematology and Oncology, Nagoya University Graduate School of Medicine, Nagoya 466-8550, Japan

[3]National Hospital Organization Nagoya Medical Centre, Nagoya 460-0001, Japan

Corresponding author:

Gyosuke Sakashita, Department of Biochemistry, Shimane University School of Medicine, Izumo 693-8501, Japan, Tel.: +81-853-20-2127; FAX: +81-853-20-2125; E-mail: gsakashi@med.shimane-u.ac.jp

Supplementary Figure 1

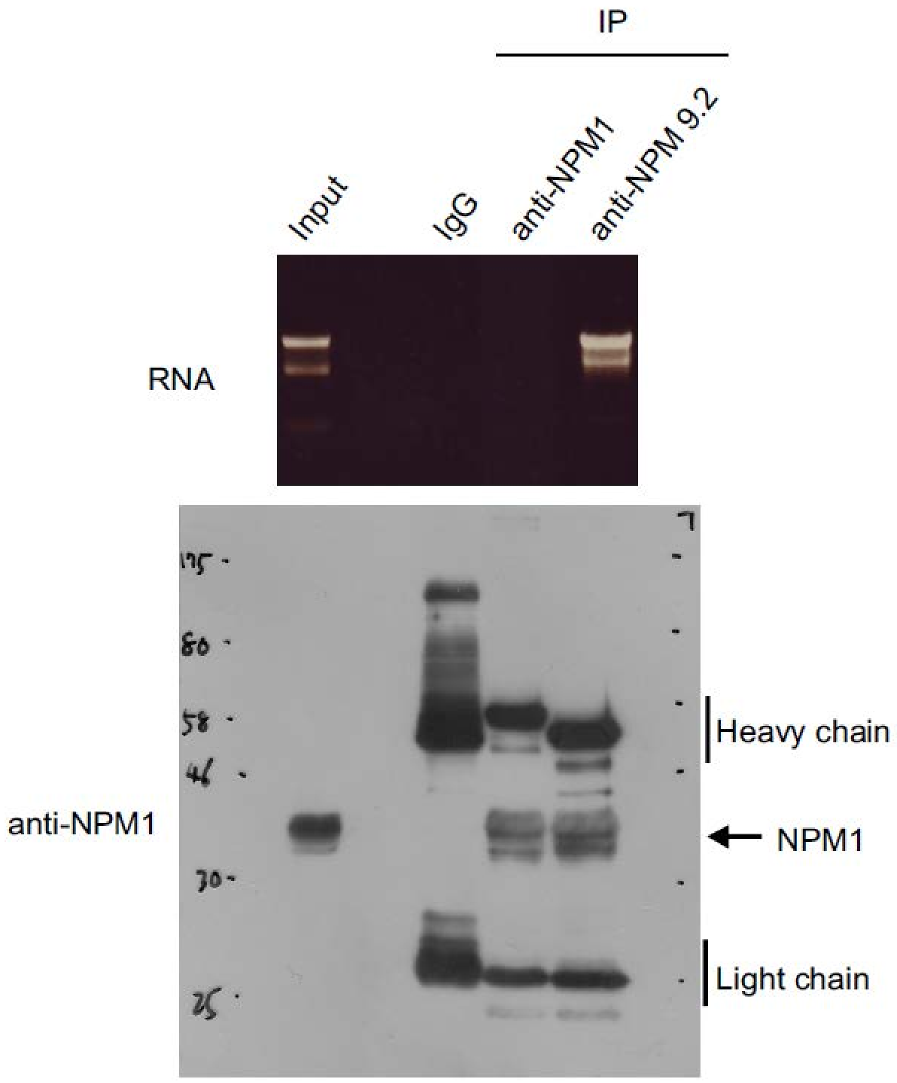

Supplementary Figure 1 - Antibody specific for NPM1 inhibits association of NPM1 and RNA. HeLa cells were lysed with PBS containing 1% Triton X-100. Immunoprecipitation was performed with anti-G196 (for control), anti-NPM1 and anti-NPM 9.2. NPM1 and RNA were detected by immunoblot and ethidium bromide staining, respectively.

Supplementary Table 1 - Primers used in this study

Primer name	Sequence (5'→3')
FHG sense primer	GGAGATCTGCCACCATGGATTACAAGGACGACGATGACAAG CTCGATGGAGGATACCCATACGATGTTCCAGATTACGCTGGA GGAGTGAGCAAGGGCGAGGA
FHG antisense primer	GGGAATTCAGGATCCGGATCTGAGTCCGGA
G196 sense primer	AGCTTCCACCATGGCAGGTTCGGATCTGGTTCCGCGTGGATC CCCAGAATTCTAAC
G196 antisense primer	TCGAGTTAGAATTCTGGGGATCCACGCGGAACCAGATCCGAA CCTGCCATGGTGGA
NPM1 sense primer	GGGGATCCATGGAAGATTCGATGGACATGGA
NPM1 antisense primer	GGGAATTCAAAGAGACTTCCTCCACTGCCA
NPM1.3 antisense primer	GGGAATTCAATGCGCTTTTTCTATACTTGCTTGCAT-3'
NPM1c antisense primer	GGGAATTCATTTTCTTAAAGAGACTTCCTC
NPM (1-128) antisense primer	GGGAATTCATTCATCTTCTGACTCTGCATC
NPM (114-219) sense primer	GGGGATCCCAGCACTTAGTAGCTGTGGAG
NPM (114-219) antisense primer	GGGAATTCATGTTGATGATGGTTTTGAGTC
NPM (190-294) sense primer	GGGGATCCGCGCCAGTGAAGAAATCTAT
NPM1(LG) sense internal primer	CCACCAGTGGTCTTAAGGGCGAAGTGTGCTTCAGGGCCAGTG CATAT
NPM1 (LG) antisense internal primer	ATATGCACTGGCCCTGAAGCACACTTCGCCCTTAAGACCACT GGTGG
Sense primer for RT-PCR	ATTGCTTCCGGATGACTGAC
Antisense primer for RT-PCR	AAGAGACTTCCTCCACTGCC

Supplementary Figure 2

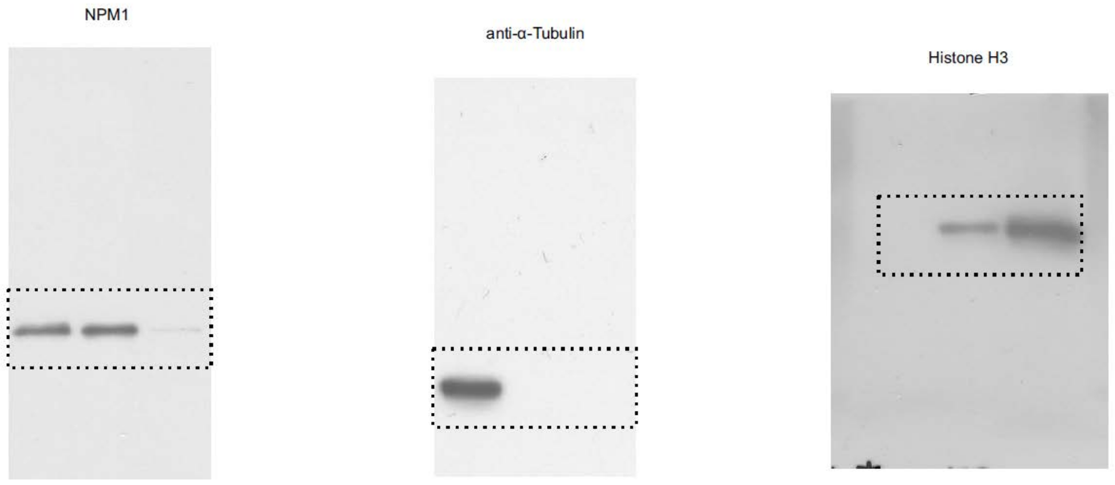

Supplementary Figure 2 – Full length images of the immunoblots presented in the Figure 2B. Black dotted line boxes indicate the cropped images used in Figure 2B.

Supplementary Figure 3

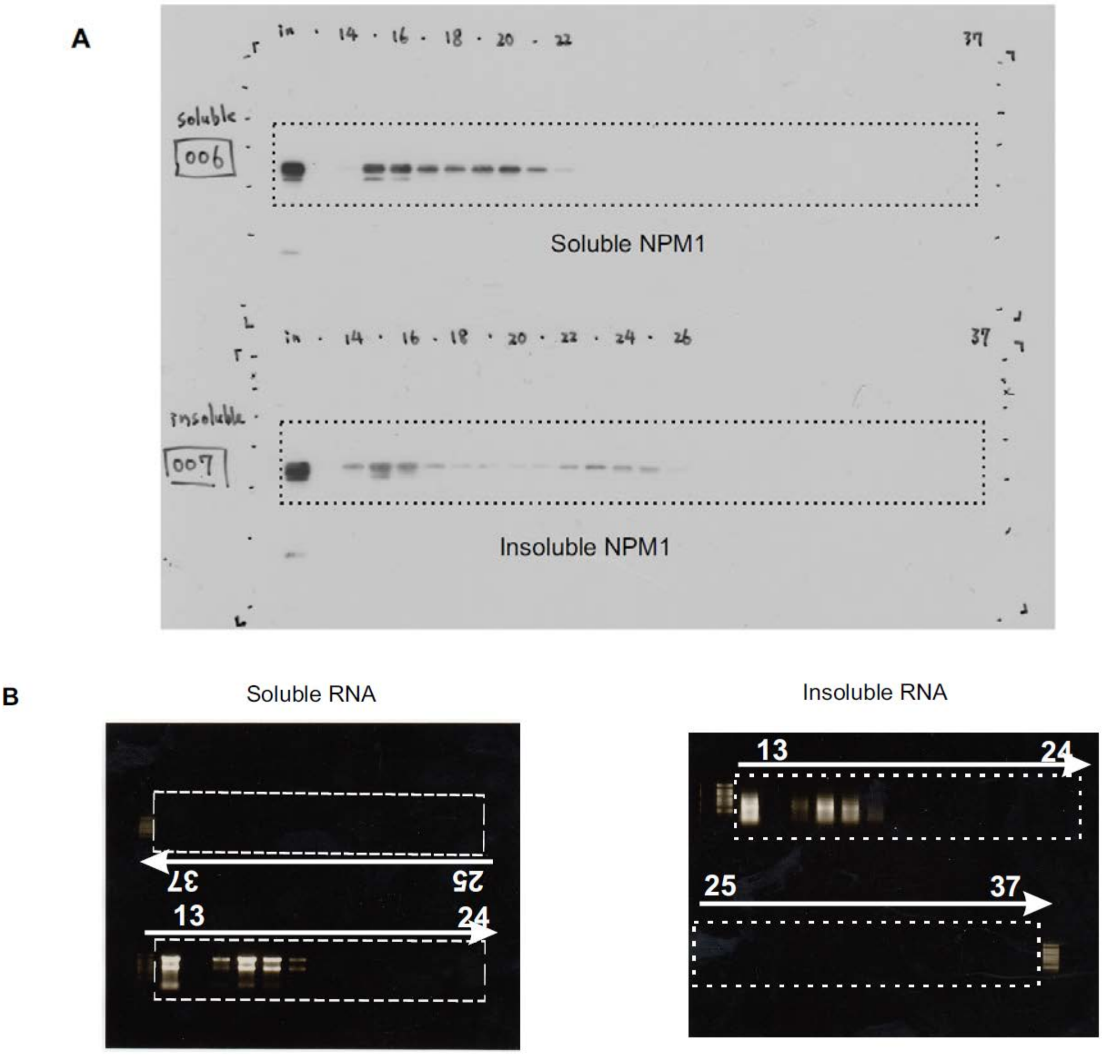

Supplementary Figure 3 – Full length images of the immunoblots and the RNA staining presented in the Figure 3A. A, Full length images of the immunoblots. Black dotted line boxes indicate the cropped images used in Figure 2A. B, Full length images of the RNA staiuning. White dotted line boxes indicate the cropped images used in Figure 2A. The arrows indicate the order of the fraction number.

Supplementary Figure 4

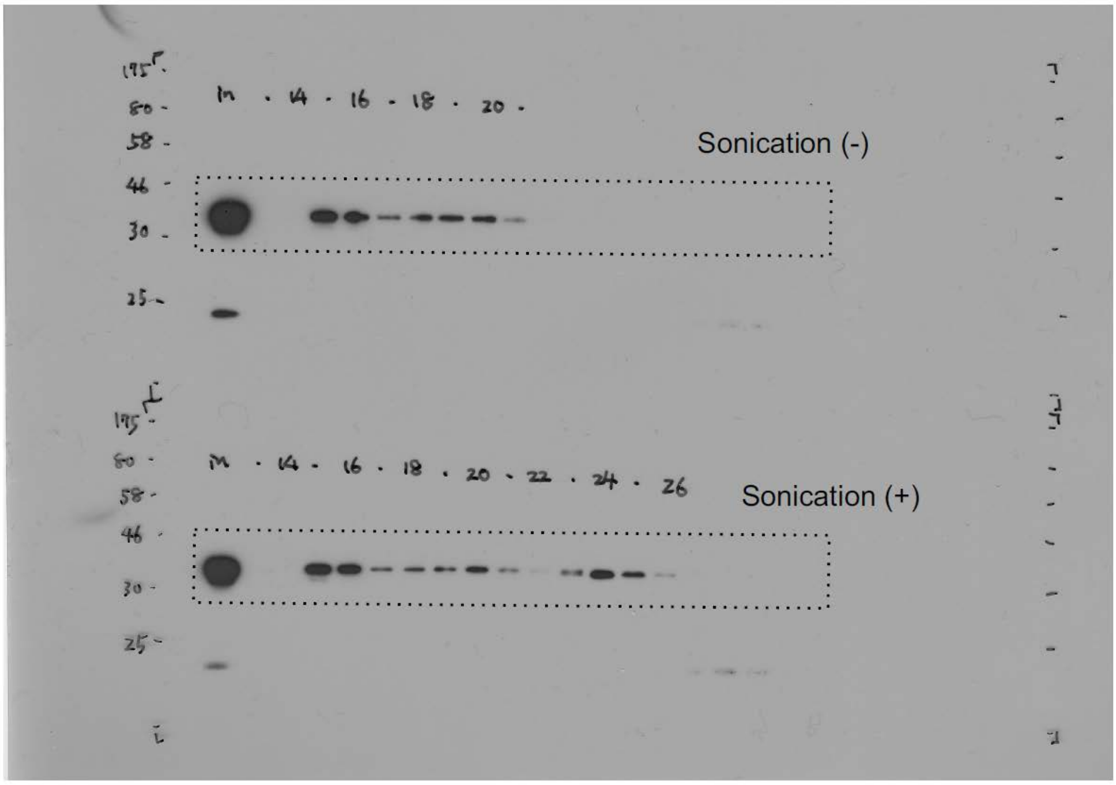

Supplementary Figure 4 - Full length images of the immunoblots presented in the Figure 3C. Black dotted line boxes indicate the cropped images used in Figure 3C.

Supplementary Figure 5

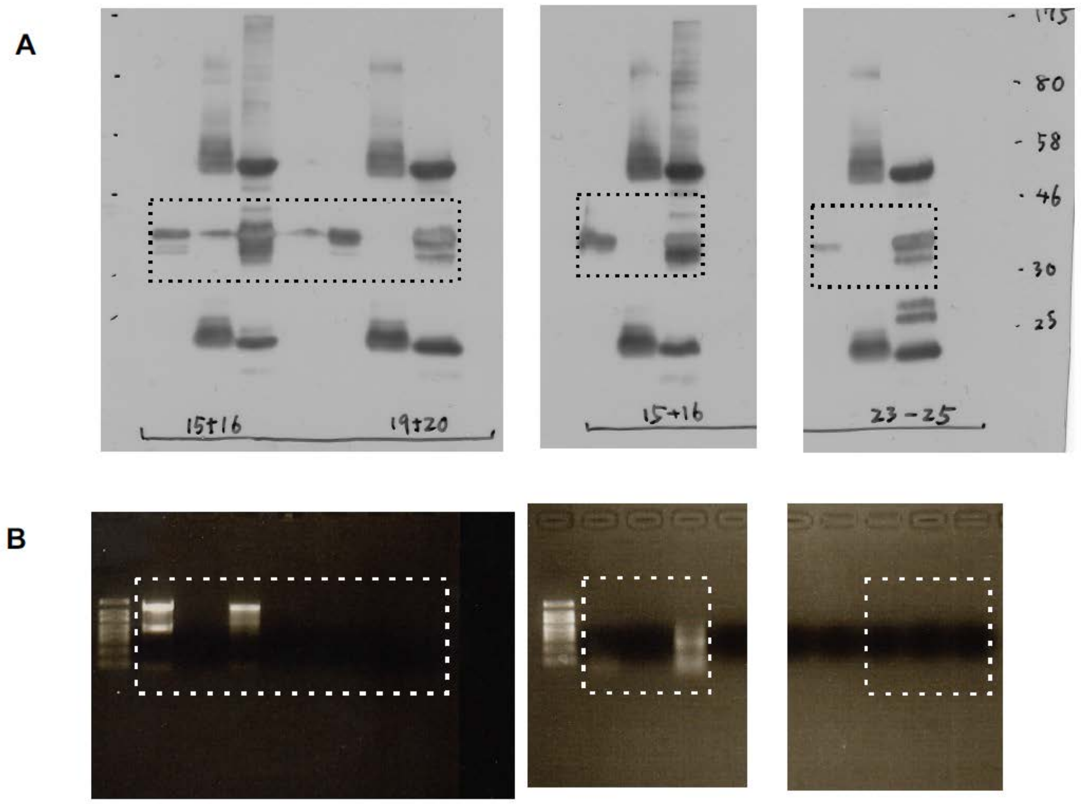

Supplementary Figure 5 - Full length images of the immunoblots and the RNA staining presented in the Figure 5A. A, Full length images of the immunoblots. Black dotted line boxes indicate the cropped images used in Figure 4A. B, Full length images of the RNA staiuning. White dotted line boxes indicate the cropped images used in Figure 4A.

Supplementary Figure 6

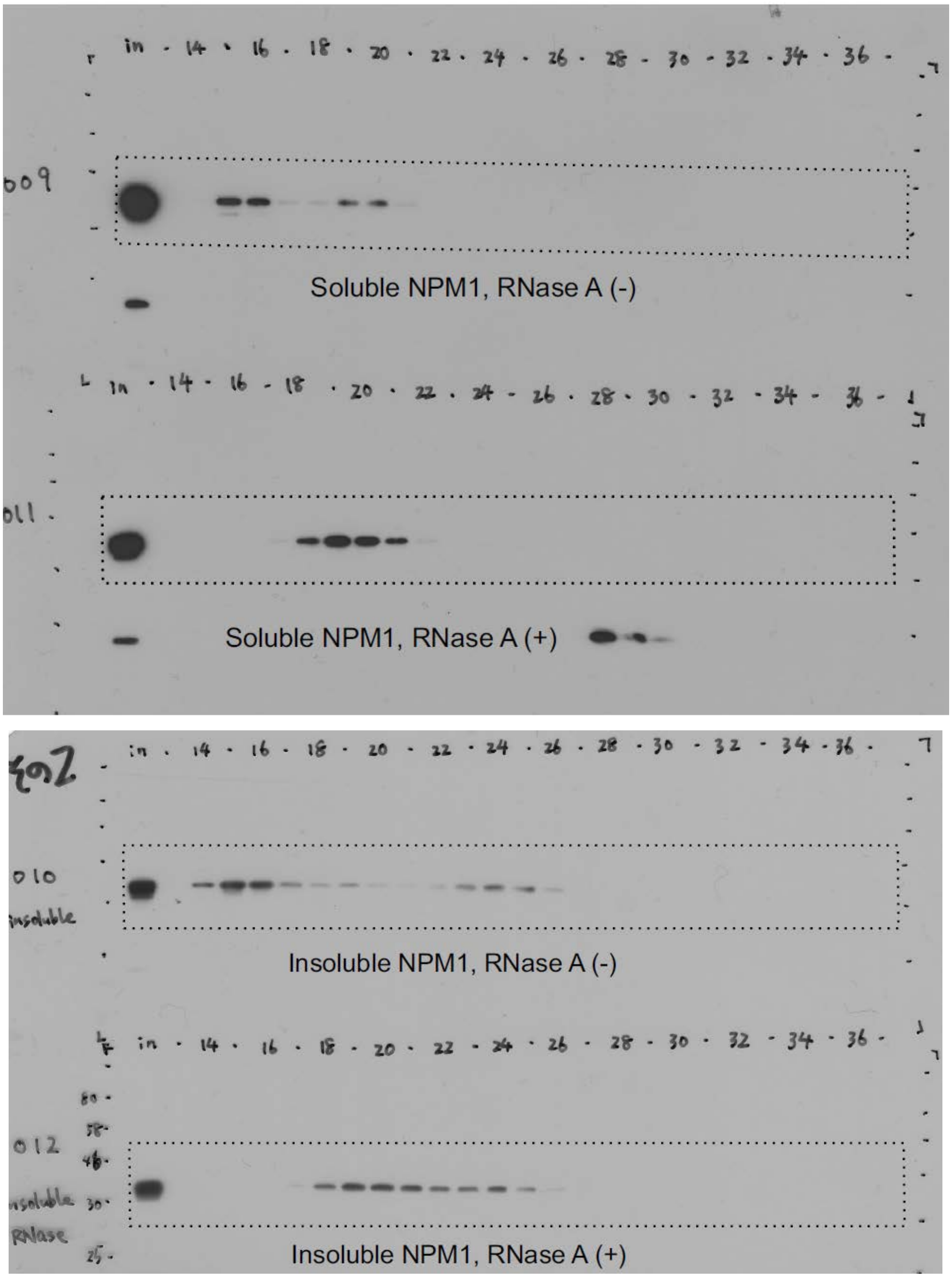

Supplementary Figure 6 - Full length images of the immunoblots presented in the Figure 4B. Black dotted line boxes indicate the cropped images used in Figure 4B.

Supplementary Figure 7

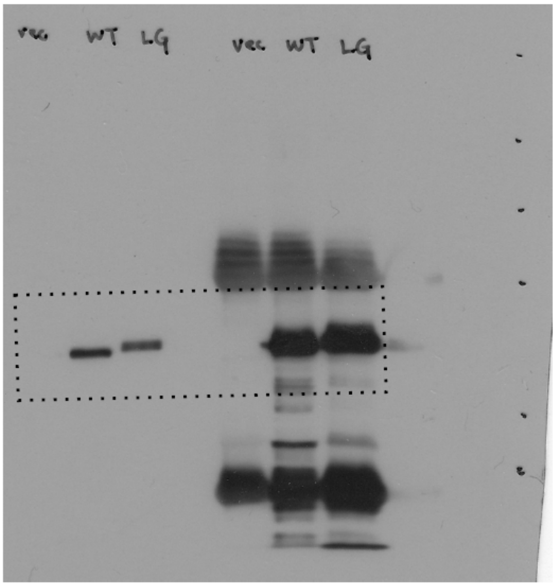

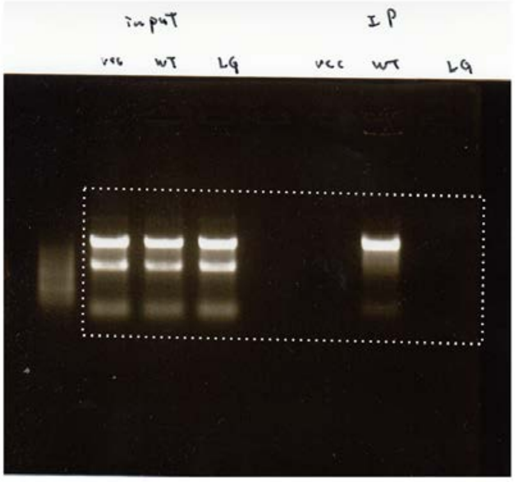

Supplementary Figure 7 - Full length images of the immunoblots and the RNA staining presented in the Figure 5A. Dotted line boxes indicate the cropped images used in Figure 5A.

Supplementary Figure 8

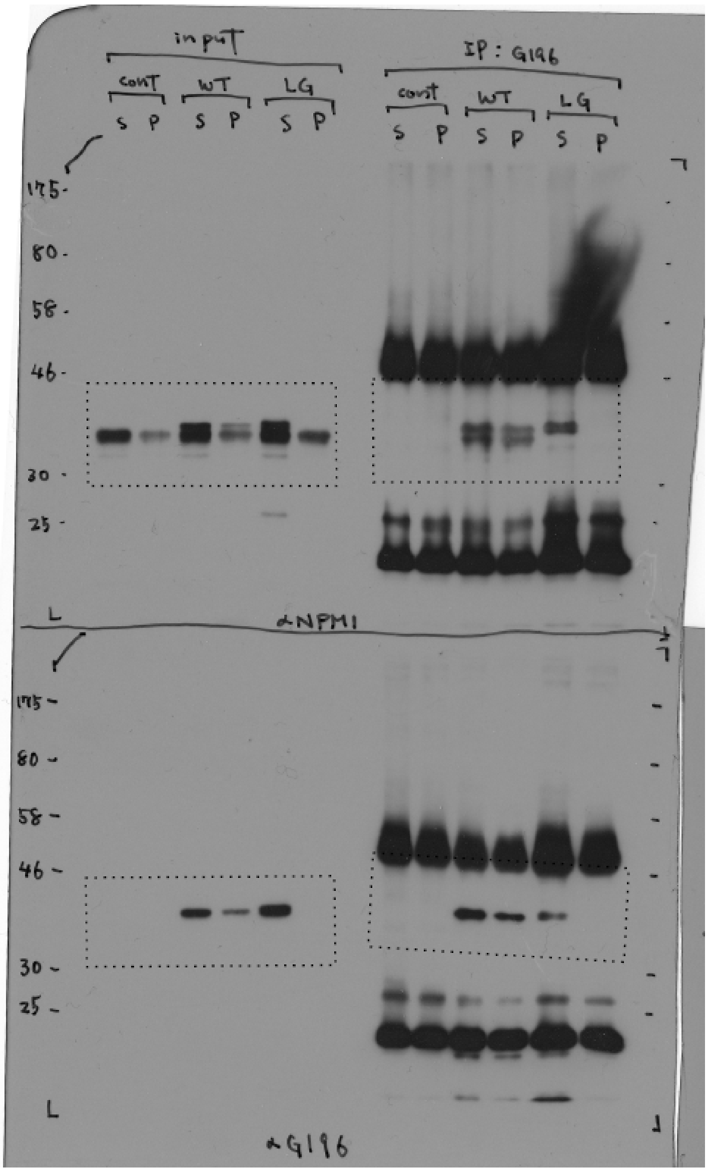

Supplementary Figure 8 - Full length images of the immunoblots presented in the Figure 5B. - Black dotted line boxes indicate the cropped images used in Figure 5B.

Supplementary Figure 9

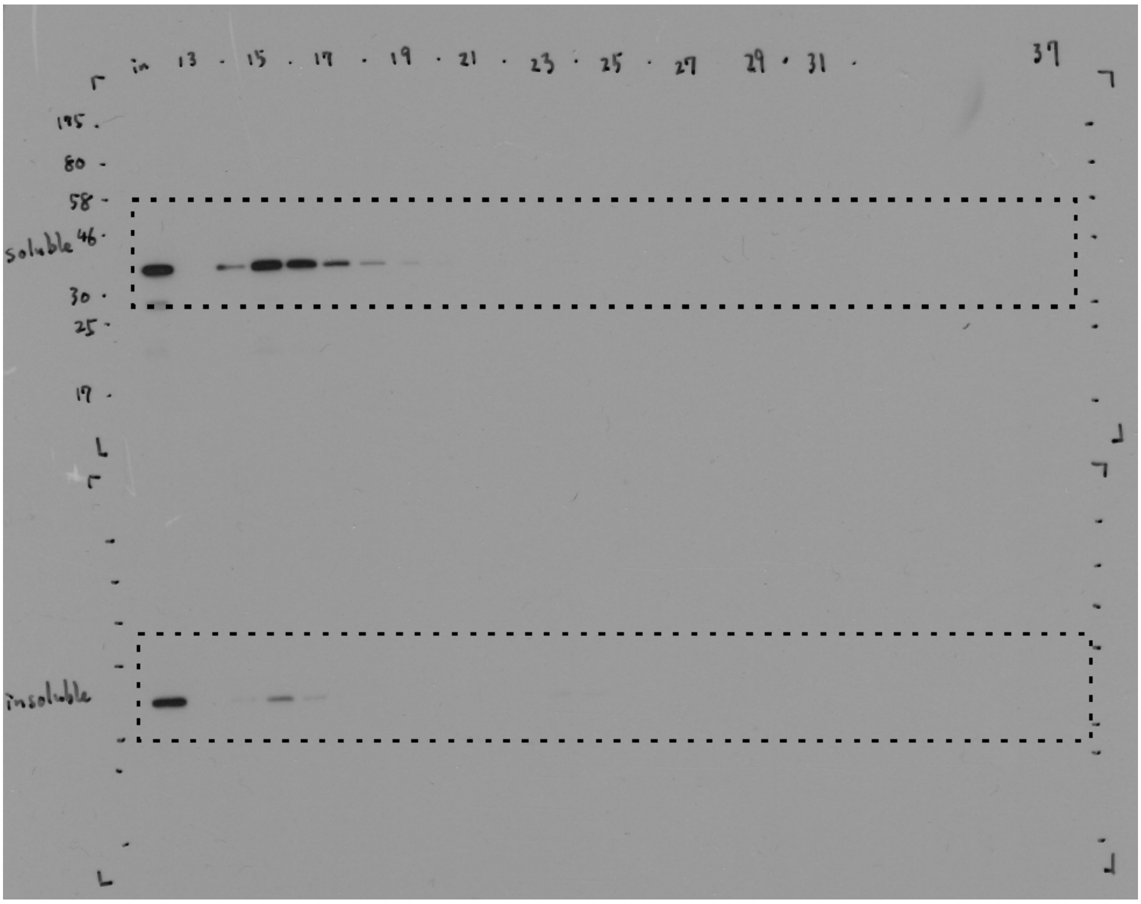

Supplementary Figure 9 - Full length images of the immunoblots presented in the Figure 5C. Black dotted line boxes indicate the cropped images used in Figure 5C.

Supplementary Figure 10

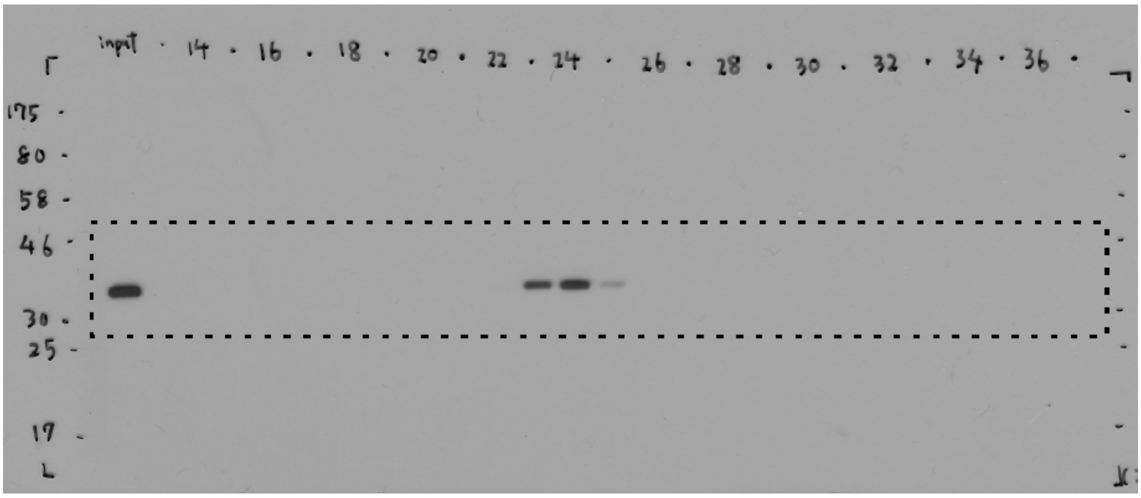

Supplementary Figure 10 - Full length images of the immunoblots presented in the Figure 5D. Black dotted line boxes indicate the cropped images used in Figure 5D.

Supplementary Figure 11

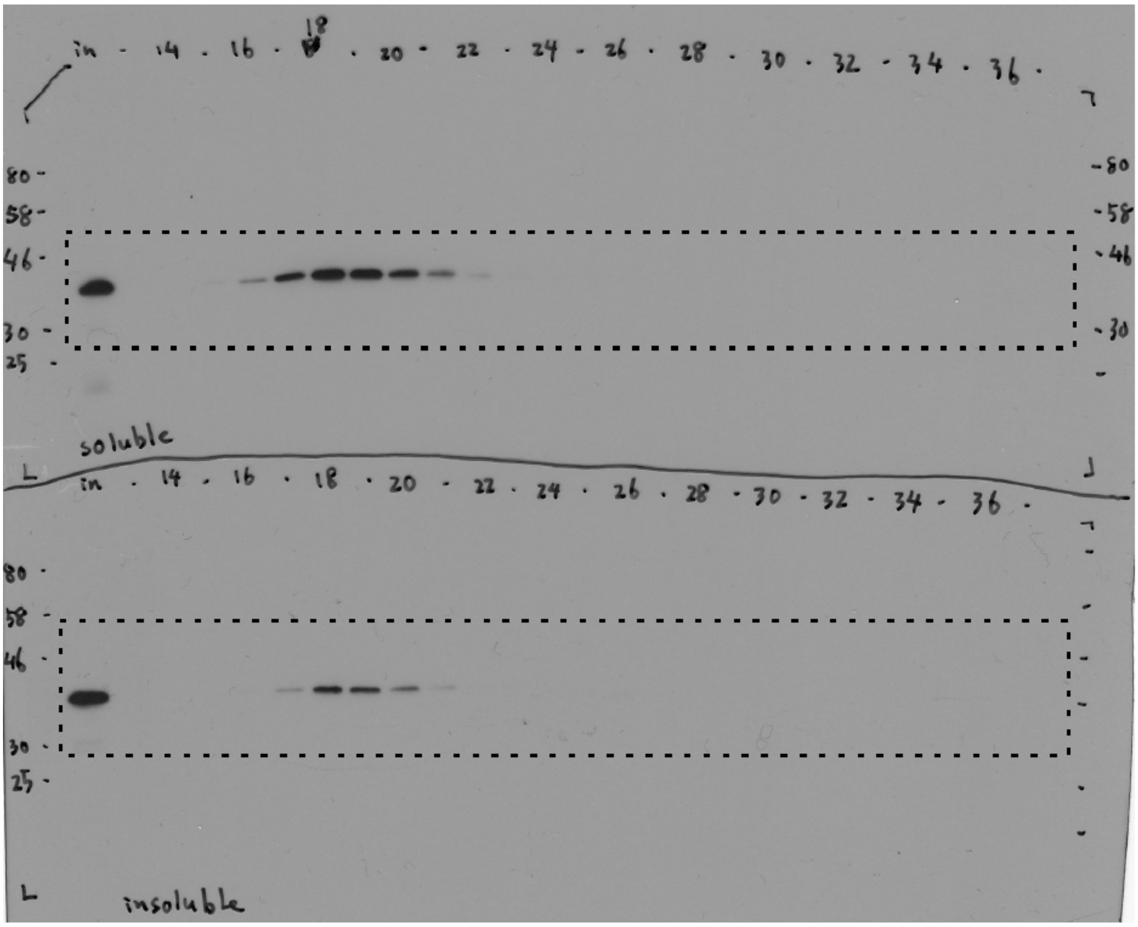

Supplementary Figure 11 - Full length images of the immunoblots presented in the Figure 5E. Black dotted line boxes indicate the cropped images used in Figure 5E.

Supplementary Figure 12

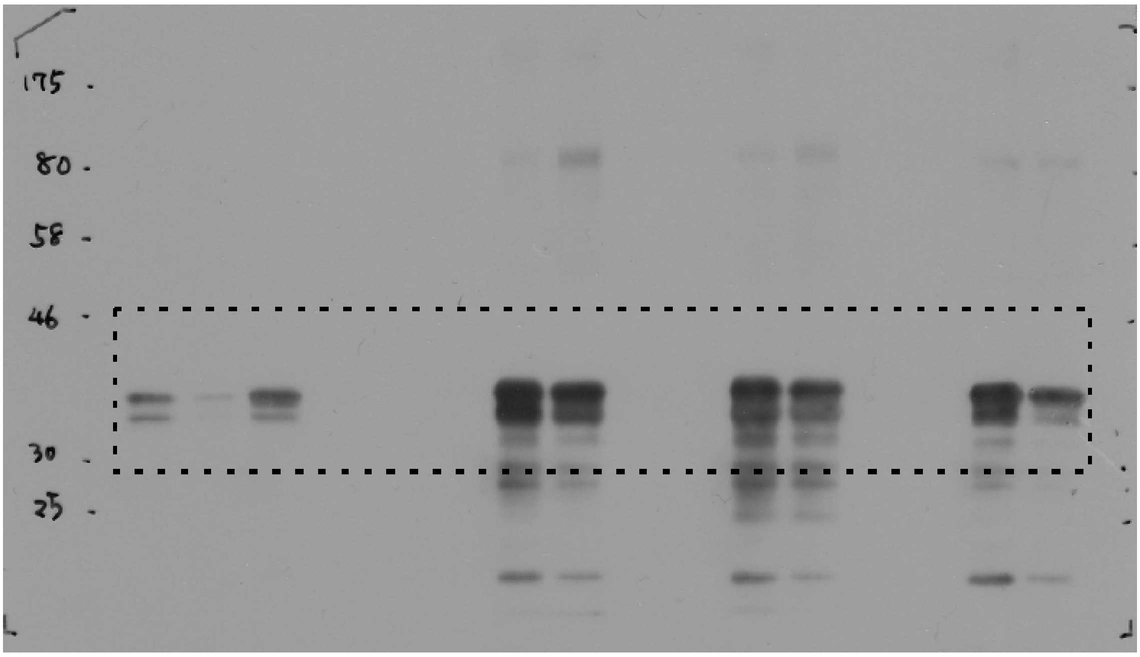

Supplementary Figure 12 - Full length images of the immunoblots presented in the Figure 7. Black dotted line boxes indicate the cropped images used in Figure 7.

Supplementary Figure 13

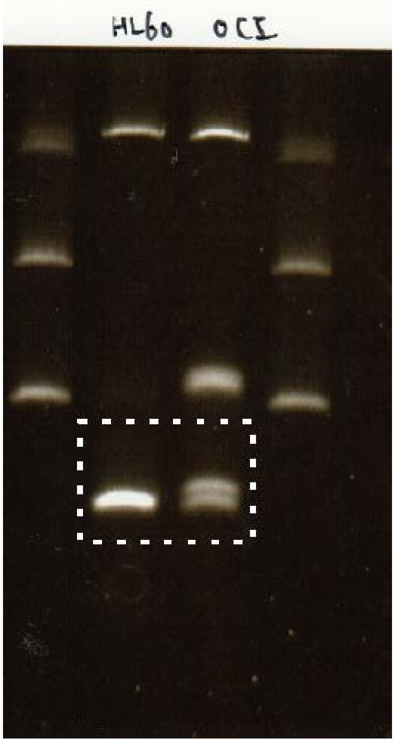

Supplementary Figure 13 - Full length images of the RNA staining presented in the Figure 8A. White dotted line boxes indicate the cropped images used in Figure 8A.

Supplementary Figure 14

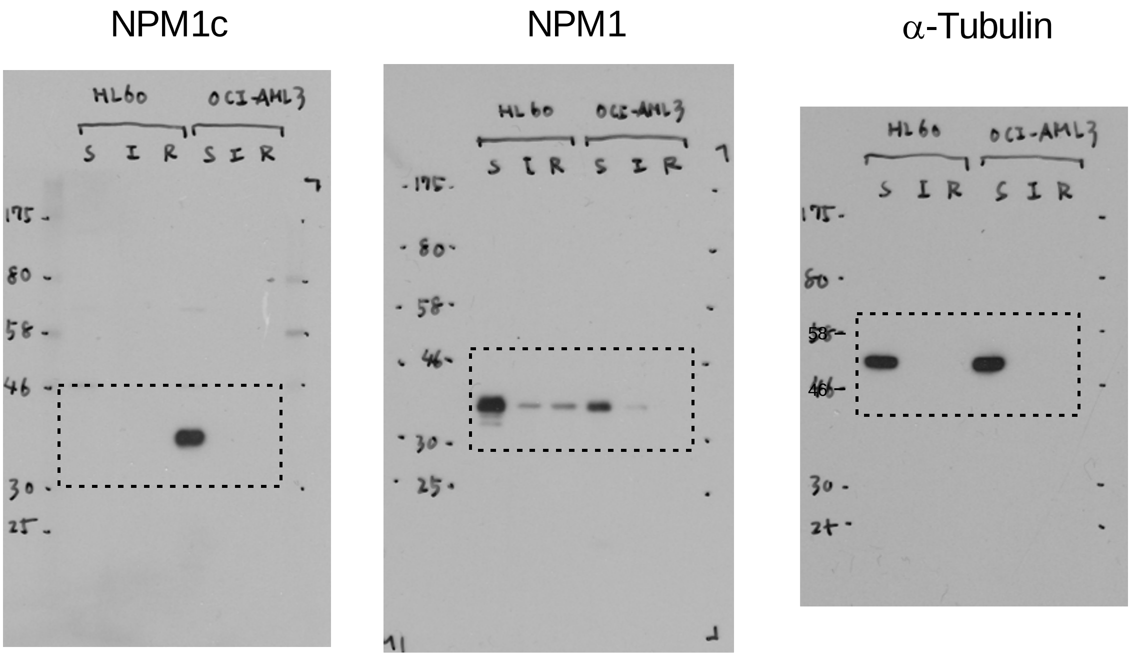

Supplementary Figure 14 - Full length images of the immunoblots presented in the Figure 8B. Black dotted line boxes indicate the cropped images used in Figure 8B.

Supplementary Figure 15

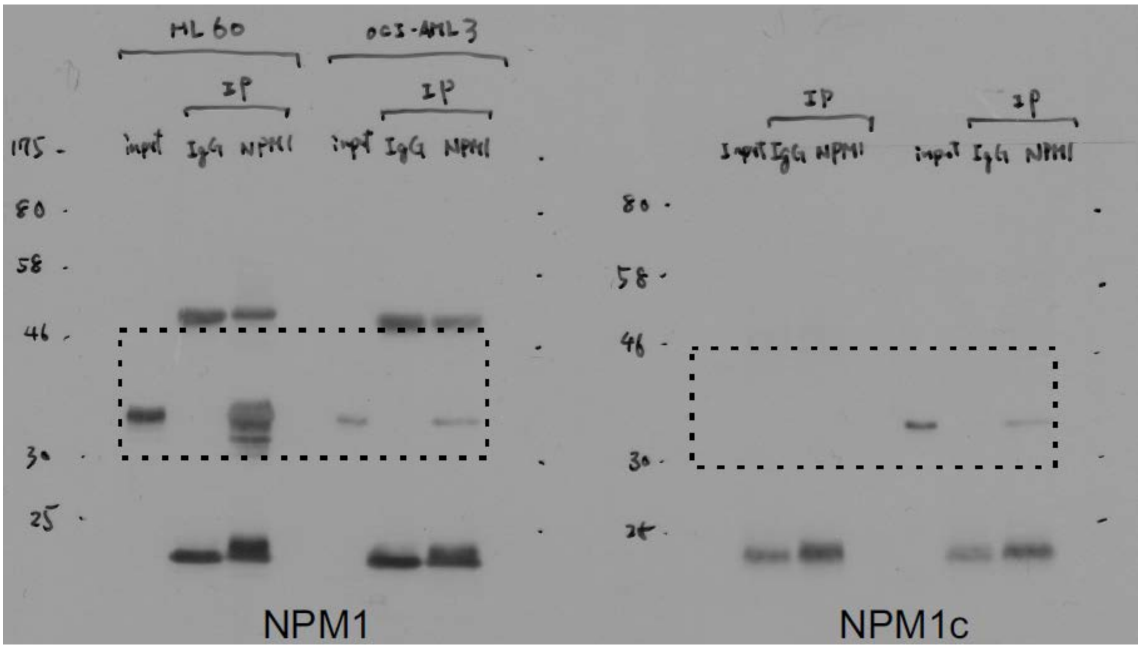

Supplementary Figure 15 - Full length images of the immunoblots presented in the Figure 8C. Black dotted line boxes indicate the cropped images used in Figure 8C.

Supplementary Figure 16

A

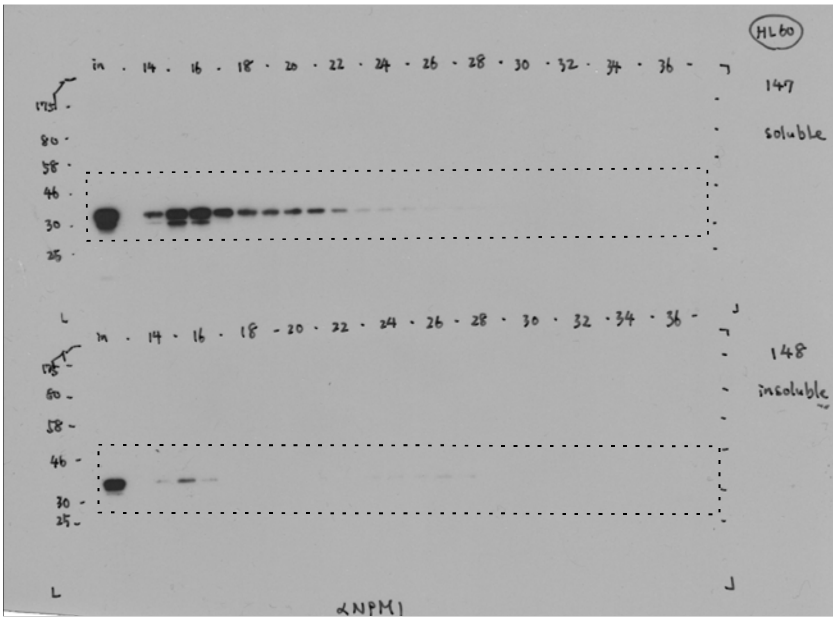

B

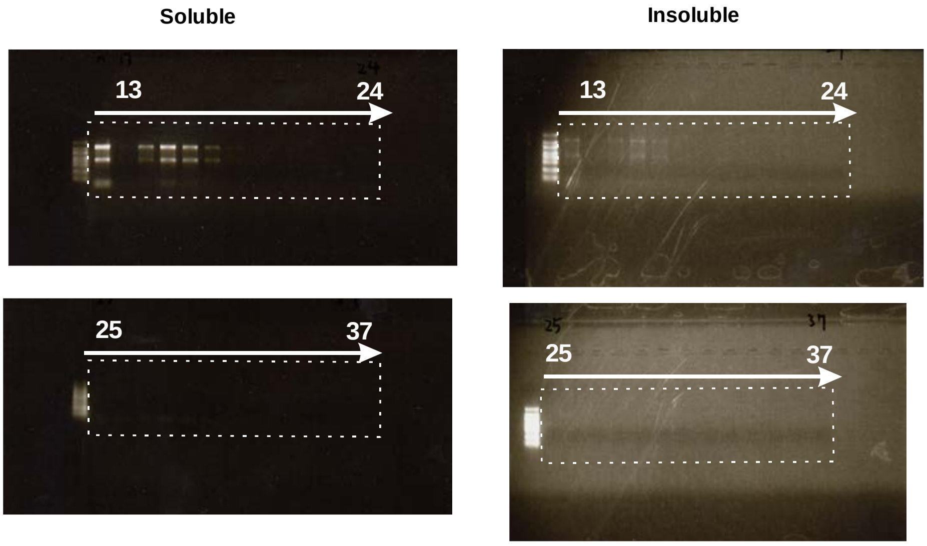

Supplementary Figure 16 - Full length images of the immunoblots and the RNA staining presented in the Figure 8D. A, Full length images of the immunoblots. Black dotted line boxes indicate the cropped images used in Figure 8D. B, Full length images of the RNA staiuning. White dotted line boxes indicate the cropped images used in Figure 8D. The arrows indicate the order of the fraction number.

Supplementary Figure 17

A

NPM1

NPM1c

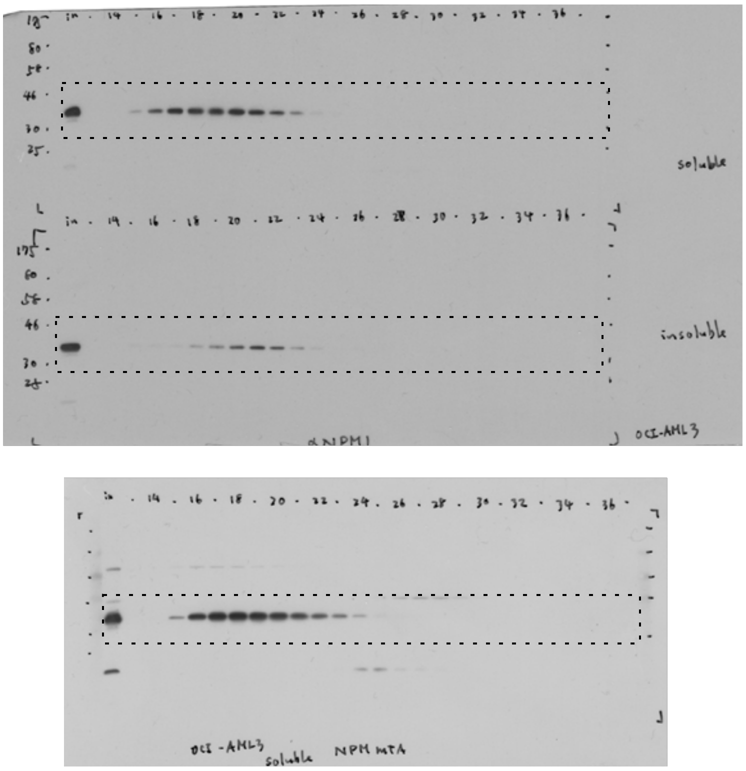

B

Soluble RNA

Insoluble RNA

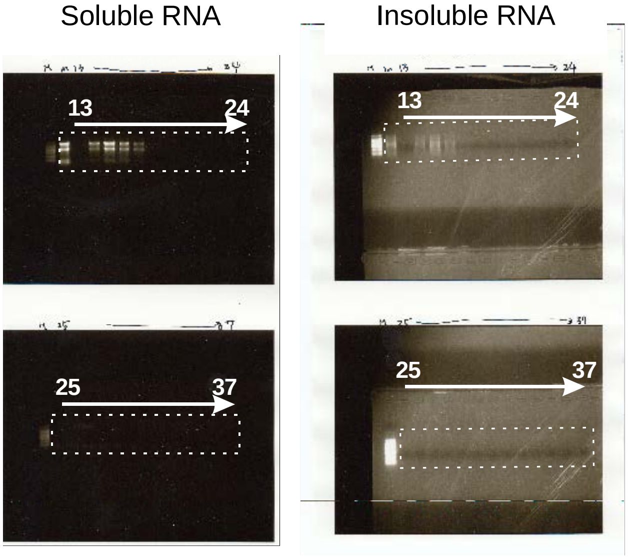

Supplementary Figure 17 - Full length images of the immunoblots and the RNA staining presented in the Figure 8E. A, Full length images of the immunoblots. Black dotted line boxes indicate the cropped images used in Figure 8E. B, Full length images of the RNA staiuning. White dotted line boxes indicate the cropped images used in Figure 8E. The arrows indicate the order of the fraction number.

Supplementary Figure 18

A

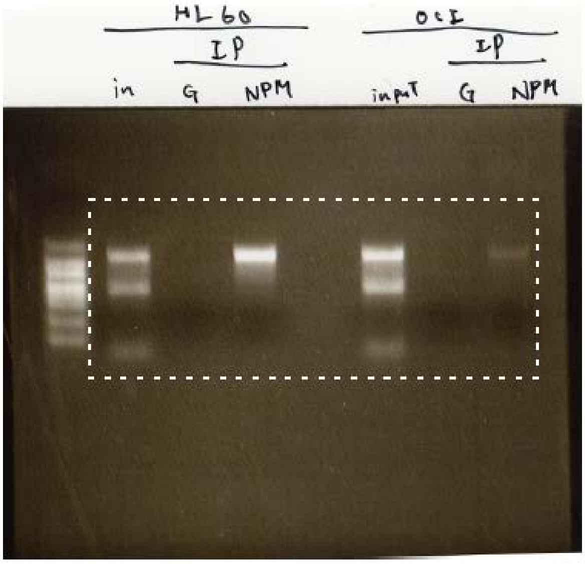

B

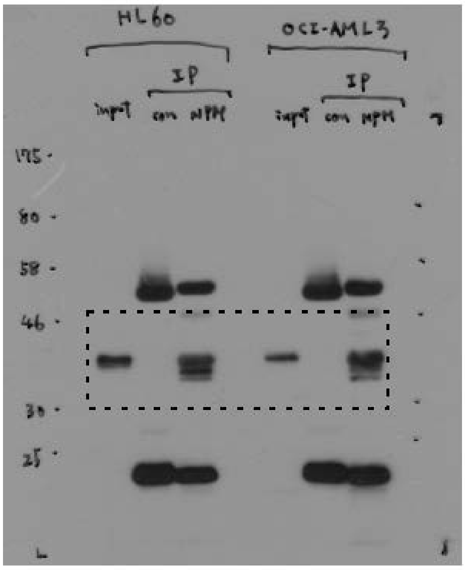

Supplementary Figure 18 - Full length images of the RNA staining and the immunoblots presented in the Figure 8F. A, Full length images of the RNA staiuning. White dotted line boxes indicate the cropped images used in Figure 8E. B, Black dotted line boxes indicate the cropped images used in Figure 8F.
